# Study of thioglycosylation in ionic liquids

**DOI:** 10.1186/1860-5397-2-12

**Published:** 2006-06-27

**Authors:** Jianguo Zhang, Arthur Ragauskas

**Affiliations:** 1 School of Chemistry and Biochemistry, Georgia Institute of Technology, Atlanta, Gerogia, 30332, USA

## Abstract

A novel, green chemistry, glycosylation strategy was developed based upon the use of ionic liquids. Research studies demonstrated that thiomethyl glycosides could readily be activated with methyl trifluoromethane sulfonate, using 1-butyl-3-methylimidazolium tetrafluoroborate as a solvent. This green chemistry glycosylation strategy provided disaccharides with typical yields averaging 75%. The ionic liquid solvent could be readily reused for five sequential glycosylation reactions with no impact on product yield.

Owing to their unique chemical and physical properties, room temperature ionic liquids (ILs) have received significant attention as alternative solvents for a host of different applications. For example, it has been reported that ILs can be used in place of conventional organic solvents in synthesis, catalysis, electrochemistry, and liquid/liquid extractions.[1] Commonly reported ILs rely on organic cations, including: tetraalkylammonium, tetraalkylphosphonium, *N*-alkylpyridinium, 1,3-dialkylimidazolium, or trialkylsulfonium species, as shown in Figure 1. A broader spectrum of anionic counter ions have been reported, including: halides, carbonates, sulfonates, tetrafluorborates, nitrates and chloroaluminates. Changes in the physical properties of ionic liquids, including their melting point, hydrophilicity, lipophilicity and polarity can be routinely accomplished by altering the nature of the ion pair or altering the nature of the alkyl group on the substituted organic cation.[2] These structural variations substantially broaden the scope and versatility of ILs applications.

**Figure 1:**
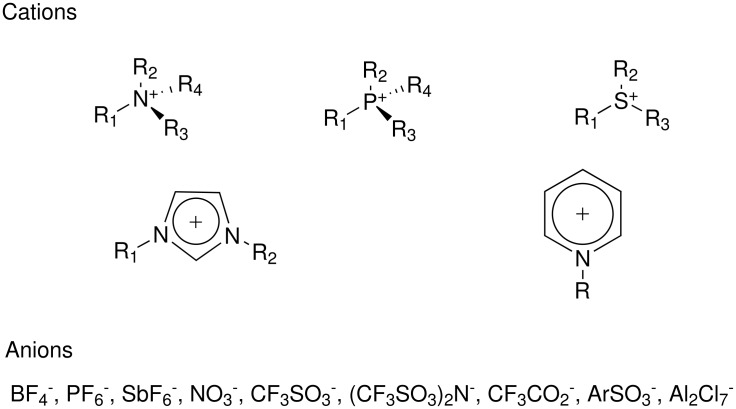
Structures of common ionic liquids.

Many chemical reactions have been carried out in volatile organic solvents that have broader environmental concerns.[3] These drawbacks have been well documented and have driven, in part, the quest for alternative solvent systems. Ionic liquids possess several attractive properties such as, no measurable vapor pressure, nonflammable, water and air stability, along with enhanced chemical and thermal stability properties. Recent reports have highlighted the potential of ionic liquids to be used as an ideal solvent for acetylation, ortho-esterification and benzylidenation of sugars,[4–6] and for certain glycosylation reactions. Sasaki et *al*. reported that the glycosidations of glucopyranosyl fluorides with assorted alcohols employing an ionic liquid and a protic acid catalyst proceeded, under mild conditions, to afford the corresponding glycosides in 54–91% yields.[7] The stereoselectivity of the glycosidation was significantly affected by the ionic liquid employed. The reactivity of glycosyl trichloroacetimidates and diethyl phosphites with alcohols in the presence and absence of lewis acids has been also recently been reported with several ionic liquids, including [bmim]PF_6_ and 1-*n*-hexyl-3-methylimidazolium trifluoromethanesulfonimidide.[8–9] These reactions typically provided over 70% yields of the corresponding glycosides or disaccharides. The intrinsic properties of the ionic liquids described above facilitate reaction work-up and recycling of the solvent.

The purpose of this investigation was to examine the potential of employing ionic liquids for the synthesis of alkyl glycoside and disaccharides via coupling of thioalkyl glycosyl donors with glycal acceptors. Alkyl glycosides and oligosaccharides are important intermediates and products in the synthesis of biologically active natural compounds and mimics. For example, tetra-*O*-acetyl-glycoside derivatives have been used in the synthesis of glycosyltransferase inhibitors and clearing agents to enhance anti-tumor activities.[10–11] In addition, alkyl glycosides possessing long alkyl chain have gained wide interest as non-ionic surfactants.[12]

A variety of reagents have been reported to promote the formation of a glycoside bond which include, classical glycosyl halides, thioglycosides, pentenyl glycosides, anomeric trichloroacetimidates and others.[13] As reviewed by Oscarson,[14] thioalkyl or thioaryl glycosyl donors have been shown to exhibit excellent selectivity and reactivity in the synthesis of oligosaccharides. Donor activation is frequently accomplished by using heavy metal salts or more directly, and efficiently, by thiophilic reagents such as methyltriflate, NBS, and DMTST.[15–16] The stereoselectivity of the glycosylation reaction is greatly influenced by the nature of the protecting group on the C_2_-hydroxyl. Neighboring group participation of the C_2_ blocking group can be used to ensure a very high degree of stereoselectivity for the glycosylation reaction.[17] Herein, we wish to report the synthesis of alkyl glycosides or disaccharides employing 1-butyl-3-methylimidazolium tetrafluoroborate (i.e. [bmim]BF_4_) as the reaction media. A number of glycosides or disaccharides were prepared by employing the coupling protocol involving thiomethyl glycosyl donors and glycosyl acceptors.

In this study, we selected methyl 2,3,4,6-tetra-*O*-acetyl-α-D-thiomannopyranoside **1** and methyl 2,3,4,6-tetra-*O*-acetyl-β-D-thiogalactopyranoside **2** as glycosyl donors to react with different glycosyl acceptors (Scheme 1). These glycosidations proceeded to smoothly give the corresponding glycosides **3a**-**3d** and **4a**-**4d** in yields ranging from 39% to 81% as summarized in Table 1. All products were consistent with literature values.[18–24] For methyl 2,3,4,6-tetra-*O*-acetyl-α-D-thiomannopyranoside **1**, the α glycosides were the major products, while for substrate **2**, the major product were β forms. However, there was a great variability in α/β ratio for 3b, 4c and 4d in this novel thioglycosylation protocol. Optimization studies indicated that a two fold molar equivalent amount of methyl triflate was required for these glycosidations to occur efficiently. Lesser amounts of methyl triflate resulted in decreased yields. For the reaction solvent, we chose 1-butyl-3-methylimidazolium tetrafluoroborate, which is a liquid at room temperature, has a low viscosity and the ability to dissolve the glycosyl donors and several glycosyl acceptors. Increasing the reaction temperature from 25°C to 75°C led to reduced product yields, and increased amounts of side products.

**Scheme 1:**
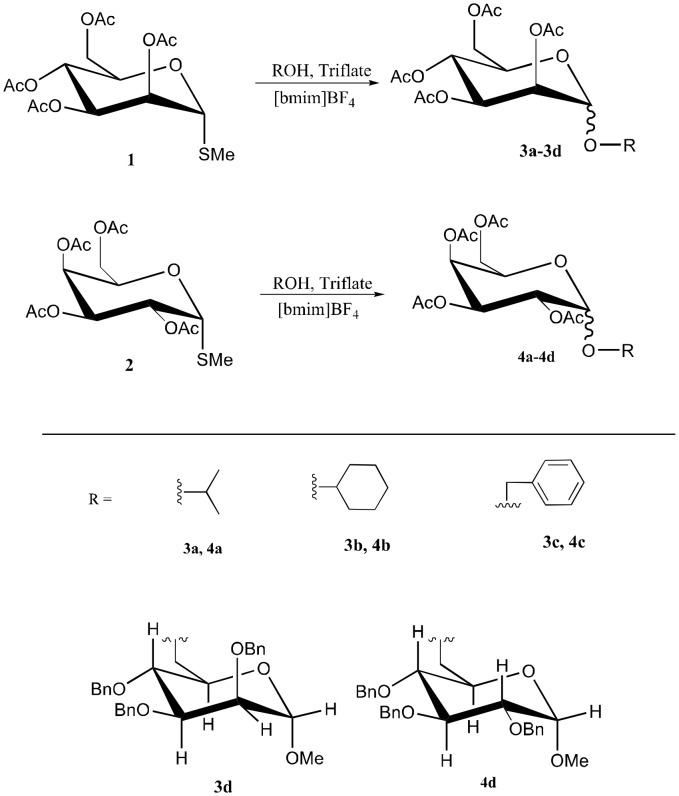
Glycosylation of **1** and **2** with various glycosyl donors.

**Table 1:** Glycosylation of **1** and **2** with various alcohols in [bmim]BF_4_ with methyl triflate.

Product	Yield (%)	α/β ratio

**3a**	78	4:1
**3b**	81	3:2
**3c**	64	1:0
**3d**	55	1:0
**4a**	76	1:8
**4b**	69	1:4
**4c**	80	5:7
**4d**	39	2:3

Other ionic liquids including 1-butyl-3-methylimidazolium hexafluorophosphate and 1-butyl-3-methylimidazolium methyl sulfate were also explored as glycosidation solvents, under the same reaction conditions. These thioglycosylation reactions in these solvents were found to be either unsuccessful or provided significantly reduced product yields. A more hydrophobic ionic liquid, 1-butyl-1-methylpyrrolidinium bis(trifluormethylsulfonyl)imide was applied for the thioglycosylation of several substrates, and the experimental data indicated that there was little reaction, indicating that 1-butyl-1-methylpyrrolidinium bis(trifluormethylsulfonyl)imide is not a good alternative ionic liquid solvent for this protocol. In the synthesis of **3c**, water was deliberately added to the reaction mixture before the addition of methyl triflate. The experimental data Table 2 indicated that the obtained yield of **3c** remained the same until one molar equivalent of methyl triflate was consumed.

**Table 2:** Glycosidation of **1** and benzyl alcohol in [bmim]BF_4_ with methyl triflate and water.

Yield of **3c** (**%**)	Methyl Triflate^1^	Water^1^

64	2.0	0.0
64	2.0	0.4
63	2.0	0.8
62	2.0	2.0
26	2.0	4.0
Reaction quenched	2.0	6.0

^1^ mMol. Note: Molecular sieves were excluded from these reactions.

This interesting stability effect may be due to the reported ability of ionic liquids to act as liquid molecular sieves.[25–26] To explore this effect, we examined the stability of methyl triflate in chloroform, dimethylsulfoxide and [bmin]BF_4_. As can be seen from Table 3, the relative hydrolysis rate of methyl triflate in DMSO is more than 100 times more reactive than in the ionic liquid. With one mole ratio of water added, the hydrolysis of triflate in DMSO was a factor of 40 times more reactive than in [bmim]BF_4_, as shown in Table 3c. These results agreed well with the thioglycosylation reactions in [bmim]BF_4_, as the addition of up to 1 molar equivalent of water did not cause dramatic change of the glycosides' yields; more than 1 molar equivalent of water resulted in a gradual decrease in yield. This indicated that the thioglycosylation of certain carbohydrates in [bmim]BF_4_ per se was not quenched with low molar equivalents of water, and [bmim]BF_4_ acted not only as reaction media, but may also performed like "molecular sieves" at the same time.

**Table 3:** a-c. The percentage of hydrolyzed methyl trifluoromethanesulfonate in DMSO, CDCl_3_, and [bmim]BF_4_.

a. Triflate/H_2_O = 0.275 mmol/0.055 mmol				

Entry	5 min	30 min	60 min	120 min

DMSO	16%	16%	17%	18%
CDCl_3_	6.4%	6.5%	6.6%	6.7%
[bmim]BF_4_	0	0	0	0.1%

b. Triflate/H_2_O = 0.275 mmol/0.111 mmol				

Entry	5 min	30 min	60 min	120 min

DMSO	24%	25%	26%	27%
CDCl_3_	6.7%	7.5%	7.8%	8.2%
[bmim]BF_4_	0	0	0	0.2%

c. Triflate/H_2_O = 0.275 mmol/0.278 mmol				

Entry	5 min	30 min	60 min	120 min

DMSO	48%	50%	53%	55%
CDCl_3_	9.5%	9.7%	9.8%	9.9%
[bmim]BF_4_	0	0.2%	0.4%	1.2%

The recyclability of [bmim]BF_4_ for thioglycosylation reactions was accessed by repeating the synthesis of **3a**, **4a** and **3c**. In brief, upon completion of the thioglycosylation reaction and extraction of the products, the ionic liquid was washed, filtered through a pad of Celite, and dried at 70°C, under reduced pressure. Following this procedure, the recovered ionic liquids were reused for the thioglycosylation reaction at least five times, without any loss in efficiency, to provide the same yields and selectivities as described in Table 1.

In summary, we have demonstrated the application of ionic liquids for the thioglycosylation methodology employing methyl triflate as an activation agent. Moreover, the results indicate that the use of ionic liquid ([bmim]BF_4_) provides a good yield and stereoselectivity with an environmentally benign protocol.

## Additional information

The experimental details can be found in Supporting Information File 1

## Supporting Information

File 1 contains experimental details.
