# Supplementary material for: Study of thioglycosylation in ionic liquids
Source: Beilstein J Org Chem. 2006 Jun 27;2:12. doi: 10.1186/1860-5397-2-12 (PMC1540430; doi:10.1186/1860-5397-2-12)
Supplement: File 1 — contains experimental details. [file Beilstein_J_Org_Chem-02-12-s001.doc]

**Typical procedure**: All the ionic liquid solvents used in this study were purchased from Aldrich-Fluka, they were dried together with 4Ǻ molecular sieves under reduced pressure at 70^o^C for several hours. The donor (0.5 mmol) and acceptor (1.0 mmol) were dissolved in ionic liquid (0.7-2.0 ml). The mixture was stirred under argon at ambient temperature, methyl trifluoromethane sulfonate (2 equiv.) was added in 30 min. After stirring at 25 ^o^C for 48 h, the mixture was quenched with TEA, and extracted with toluene (6 x 3 ml), the organic layer was then washed with brine and water, dried over Na_2_SO_4_ and the combined extracts were removed in *vacuo*. Purification of the residue by column chromatography gave the glycosides.
